# A Hydro-Economic Model for Water Level Fluctuations: Combining Limnology with Economics for Sustainable Development of Hydropower

**DOI:** 10.1371/journal.pone.0114889

**Published:** 2014-12-19

**Authors:** Philipp Emanuel Hirsch, Sebastian Schillinger, Hannes Weigt, Patricia Burkhardt-Holm

**Affiliations:** 1 Research Centre for Sustainable Energy and Water Supply, University of Basel, Basel, Switzerland; 2 Program Man-Society-Environment, Department of Environmental Sciences, University of Basel, Basel, Switzerland; 3 Department of Biological Sciences, University of Alberta, Edmonton, Canada; University of Delhi, India

## Abstract

Water level fluctuations in lakes lead to shoreline displacement. The seasonality of flooding or beaching of the littoral area affects nutrient cycling, redox gradients in sediments, and life cycles of aquatic organisms. Despite the ecological importance of water level fluctuations, we still lack a method that assesses water levels in the context of hydropower operations. Water levels in reservoirs are influenced by the operator of a hydropower plant, who discharges water through the turbines or stores water in the reservoir, in a fashion that maximizes profit. This rationale governs the seasonal operation scheme and hence determines the water levels within the boundaries of the reservoir's water balance. For progress towards a sustainable development of hydropower, the benefits of this form of electricity generation have to be weighed against the possible detrimental effects of the anthropogenic water level fluctuations. We developed a hydro-economic model that combines an economic optimization function with hydrological estimators of the water balance of a reservoir. Applying this model allowed us to accurately predict water level fluctuations in a reservoir. The hydro-economic model also allowed for scenario calculation of how water levels change with climate change scenarios and with a change in operating scheme of the reservoir (increase in turbine capacity). Further model development will enable the consideration of a variety of additional parameters, such as water withdrawal for irrigation, drinking water supply, or altered energy policies. This advances our ability to sustainably manage water resources that must meet both economic and environmental demands.

## Introduction

The development of hydropower is a controversial issue. New dams use water as a renewable energy source and can help to limit emissions. The storage of water in reservoirs also alleviates supply problems of drinking water, provides water for irrigation, and improves flood control [1]. But new dams also come with far-reaching ecological consequences. The damming of rivers has altered the biodiversity and functioning of fluvial ecosystems on a global scale [2]. Dams impair the natural flow regime of rivers and decrease the longitudinal and latitudinal connectivity of aquatic ecosystems; altered nutrient cycling, caused by flow changes and impaired species movements, caused by blocked migration routes, are just two major consequences. The creation of reservoirs also affects terrestrial ecosystems, e.g., by the submergence of plant biodiversity in newly flooded valleys [3], [4].

Despite the detrimental effect of a dammed lake's transformation into a reservoir, it continues to be an ecosystem. This reservoir ecosystem, albeit shaped by humans, hosts biota that respond to a hydropower operation. One predominant change caused by hydropower operation is the shift from natural to artificial water level regimes. For example, in water and mountain-rich European countries more than 30% of all natural lakes lost their natural water level regime. In 95% of the cases the natural regime gave way to water levels governed by hydropower operations [5].

The level of water has particularly profound influence on ecosystem processes in lakes, because it affects the littoral zone [6]. The littoral zone, whose extent is governed by the lake level, is of disproportionate importance to the food web of lakes [7]. Littoral plants and algae account for a major part of the primary production of lakes (i.e. biomass formation through photosynthesis). Littoral primary production is particularly important in clear high-altitude lakes with low nutrient concentration. For instance, the clearer and less nutrient-rich the water, the more solar radiation reaches the inundated littoral zone, and the more primary production of the lake ecosystem takes place in the littoral [8]. Lakes that are less rich in nutrients but have a functioning littoral zone can thus support a much higher biomass and diversity of species [9]. Water levels fluctuate naturally and can be important triggers of fish and other species' life-cycles, e.g., by creating suitable spawning or refuge habitat in the form of freshly flooded or beached areas [10]. Water level fluctuations (henceforth WLF) can also alter the redox potential over sediments and the gas exchange between the lake and the atmosphere. For example, WLF affect the ebullition of methane from littoral sediments, alter further biochemical water parameters, and even influence water clarity in the whole lake [11], [12].

The production of hydropower in dammed rivers and lakes leads to WLF that differ substantially from natural WLF. Especially the amplitude of WLF is affected by hydropower production. Natural WLF are in the range of few centimeters to a maximum of 3 m, whereas anthropogenic WLF typically show much broader ranges: from a few meters up to 100 m [12]. In Table 1 we compiled data on WLF caused by water management of reservoirs and WLF from pristine lakes with a natural water level regime. The difference between the two types of WLF is especially illustrative, where data on WLF before and after damming is available. The construction of a dam typically leads to WLF amplitudes increasing five- to almost tenfold (Table 1). This drastic change in the amplitude of WLF results from the transition of water levels governed by hydrological processes, to water levels governed by hydropower operation. The few well-described cases of what happens in the event of a dam removal, epitomize the ecological impacts of dams and their fluctuating water levels: upon removal of dams and restoration of pristine water levels, biodiversity and lake ecosystem functioning will largely return to pre-dam levels [13]. The approximately 40,000 large reservoirs in the world, however, are here to stay. Their number will increase rather than decrease in the future [3], [4]. Clearly, as B. Moss put it, “We must make the best of them,” and that includes managing WLF [1]. To achieve a sustainable management and hydropower development, we need a methodology, one that allows an assessment of how different hydropower operation schemes will translate into changes in WLF. The need for a method to assess WLF in the context of hydropower is all the more pressing, because the development of hydropower will be amongst the most profound stressors for aquatic ecosystems in the future [14], [15].

**Table 1:** Differences between natural and anthropogenic water level fluctuations.

Anthropogenic water level fluctuations	Natural water level fluctuations	Study system, location, reference
6 m^*^	2.5 m ^§^	Lake Kemijärvi, Finland [5]
10 m^*^	2 m ^§^	Lake Suorvajaure, Sweden [5]
9 m^*^	1 m ^§^	Lake Øyeren, Norway [5]
10 m^*^	≤ 1 m ^§^	Loch Quoich, Scotland [5]
	1–3 m	Lake Constance, Germany [6]
10 m		Serra Serrada Reservoir, Portugal [56]
	1 m	Lake Van, Turkey [57]
5–140 m		100 reservoirs, Norway [5]
	2 m	16 lakes, Canada [58]
≤ 1–35 m		563 reservoirs, Sweden [5]
	≤ 1 m	Lake Zuerich, Switzerland [59]
15 m		Saidenbach Reservoir, Germany [60]
	2 m	Lake Balaton, Hungary [6]
≤ 1–74 m		123 reservoirs, Norway [61]
50 m		Mattmark Reservoir, Switzerland [15]

List of different annual maximum amplitudes (+/− 0.5m) of natural WLF and ‘anthropogenic’ WLF caused by hydropower operations or a combination of hydropower and other water usages. If available, data on WLF before^ §^ and after ^*^ dam construction are provided.

In reservoirs, WLF follow the operating scheme of the power plant, which discharges water through the turbines to produce energy. Data on WLF caused by this operation of hydropower plants are hard to obtain. Operators record water level data, but have little incentive to make their reservoir's WLF data and hence operation schemes available to any other party. Publishing such data could make the corporate strategy accessible for competitors, which might entail competitive disadvantages. This creates the need for models that indirectly estimate water levels and hence WLF. In a purely natural context of pristine lakes, modelling WLF is possible with ever increasing accuracy. For example, using elaborate hydroinformatics, Noury et al. [16] modelled the hydrologic drivers of WLF in Umuria lake, Iran, with great precision. In reservoirs, however, it is energy production that mainly drives WLF and not hydrological processes [12]. This calls for an integrative modelling approach that combines hydrological with economic drivers of reservoir operation and hence WLF. Hydrological models that do not consider the operating scheme will be unsuitable to capture the development of WLF in the context of hydropower operations. Recently, hydro-economic models have been developed to assess and balance conflicting water demands on a basin scale. For example, Kimaite [17] presented a model that can be used to simulate how growing water demand can be met with a change in reservoir operation policy in Georgia, USA. In a different approach, Luwesi et al. [18] combined a monetary inventory model with hydrogeologic information on water and land use. Luwesi et al.'s model was used to evaluate the efficiency of water withdrawal from the Muooni Dam, Kenya, under different scenarios of rainfall.

**Figure 1:**
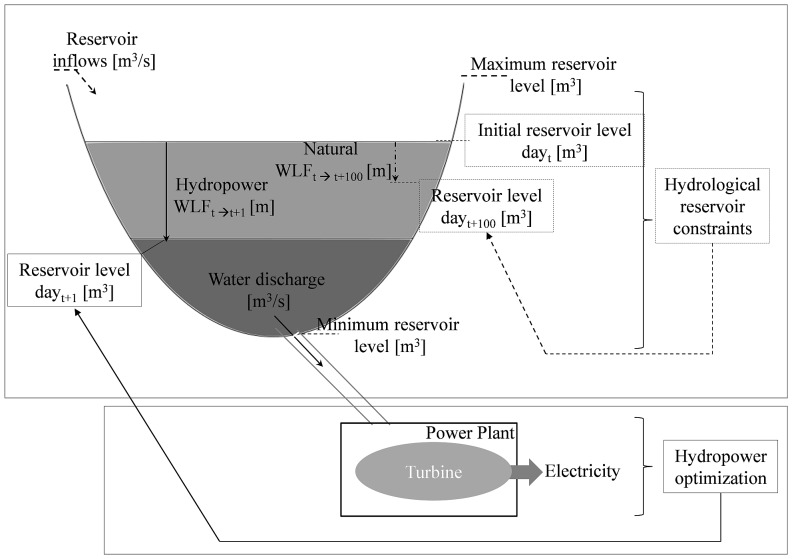
Water level fluctuations in a hydropower reservoir described by hydrological and economic parameters. Conceptual diagram of water level fluctuations (WLF) and their assessment via a hydro-economic model. Dashed arrows indicate an indirect and the solid arrows a direct influence. The difference between natural and anthropogenic WLF (see main text and table 1 for more details) is demonstrated as the amplitude of WLF within two fictional time periods where t denotes the time period (_t+100_ and _t+1_ meaning 100 and one day, respectively, after day one).

In the present study, we advance these approaches by combining hydrological drivers of water levels in reservoirs with the assumption, that reservoirs are operated to maximize profit. The aim of our study is to present a model that accurately describes WLF and hence processes in the reservoir, above the dam. This contrasts previous models that aim for a description of water supply and demand below the dam (e.g. [17], [18]). The resulting hydro-economic model allows for an assessment of WLF in reservoirs and promotes our understanding and scientific basis for reservoir management. The context of how WLF result from hydropower operation is illustrated schematically in Fig. 1: the hydrological constraints of the reservoir, such as maximum storage volume and inflow parameters, set the boundaries. Within these boundaries the operation of a power plant seeks to maximize profit by optimally discharging water through the turbines. Based on this concept, we develop a basic model which is generic and can be applied to any reservoir. To specify and validate the model, we apply it to a case study. Using a Swiss lake managed for hydropower, we test the match between modelled water levels and real water levels and calculated a reference scenario as a benchmark case (Sc1). We continue to illustrate the general validity of our model by comparing its output to compiled data from 85 other alpine reservoirs. In a further step, we test the model's applicability to assess WLF under changing climate and different political constraints. We realized this test by comparing the specific reference scenario for one lake (Sc1) with two possible future developments of water management: a climate change scenario (Sc2) which assumes changes in the inflow into the reservoir, and a price change scenario (Sc3) which assumes a changing development of the energy market and hence price fluctuations. We conclude that our model can serve to inform a more sustainable development of hydropower.

## Materials and Methods

### Generic model, defining endogenous parameters

#### Economic model development: describing the system of a hydropower reservoir

The optimal operation of a reservoir is to discharge water when electricity prices are high and retain water when prices are low (Fig. 1). Water that has been discharged through the turbines to generate energy is not available for later turbination. Thus, the problem can be stated as to find out the water discharges, at all periods of the time horizon, t  =  1,…, T, that optimize an objective function, subject to constraints. Capturing this rationale, we will present a generalized model framework in the following. We will restrict the model to those elements necessary to capture the basic economic and physical aspects. Endogenous model variables are presented in upper case, whereas exogenous parameters are given in lower case. The exogenous parameters can be adjusted to simulate varying natural or market conditions (see Sc1–Sc3). Potential extensions and inclusion of more detailed parameters into the model are introduced in the discussion section and further details can be found in the supporting information (S1 File).

To capture the profit maximization rationale of hydropower, the model needs to include *i)* the economic framework of plant operation, *ii)* the physical bounds of the operation (i.e. hydrological parameters such as inflow, (Fig. 1), *iii)* the operational constraints (such as turbine capacities).

- The objective optimization function to be maximized can be expressed as:

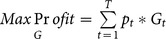


with *p_t_* being the energy price at time *t* and *G_t_* the energy generated in time *t*. Equation (I) basically represents the monetary value of the discharged water, which is to be maximized in the given time horizon *T*. Water essentially is a common property, and as such a free resource. All cost blocks of hydropower plants are mostly independent from the actual operation (i.e. labor costs need to be paid regardless of hourly output). Hence, the cost element can be omitted from the profit maximization.

The objective function is subject to constraints of two kinds: equality constraints and inequality constraints or simple bounds on the variables. They can be formulated as:

II. The power generation, which can be expressed as:

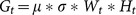
; 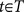


The amount of energy generated *G_t_* in time *t* is a function of the water discharge *W_t_* through the turbines at time *t*, the average head of reservoir *H_t_* at time *t* and an operational efficiency factor *σ.* The conversion factor *µ* converts the specific water flows *W* (in m^3^ s^−1^) into electricity *G* (in MWh).

III. Inflows and outflows over time follow the water balance equation of the reservoir, such that:

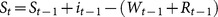
; 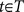


The storage level *S_t_* in time *t* depends on the previous time *t-1* storage level *S_t-1_*, the natural inflows *i_t-1_* between *t* and *t-1*, the amount of turbinated water *W_t-1_* and the residual flow *R_t-1_* at the time *t-1*. The residual flow is a legally binding restriction, typically in form of a minimum discharge or spillage. Power plant operators are obliged to provide this certain amount of water to secure minimal flows in waters downstream of the power plant [19].

Water storage is bound by the upper and lower storage level given by the reservoir capacity such that:


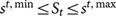
; 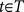


where s*^t,min^* is the minimum storage volume and s*^t,max^* the maximum storage volume.

IV. Discharges for hydropower production are bound by turbine capacity such that:

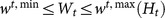
; 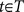


where w*^t,min^* is the minimum discharge and w*^t,max^* the maximum discharge, which can be considered dependent on head (*H_t_*).

V. Minimum discharges are bound by residuals flows, that must not be undercut by legal obligations (see III).

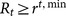
; 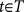


r*^t,min^* is the minimum residual water flow restriction at time *t*.

To solve the resulting quadratic model, a wide array of mathematical software can be utilized. We apply the *quadprog.m-function* in Matlab.

#### Translating the economic function into WLF

**Figure 2:**
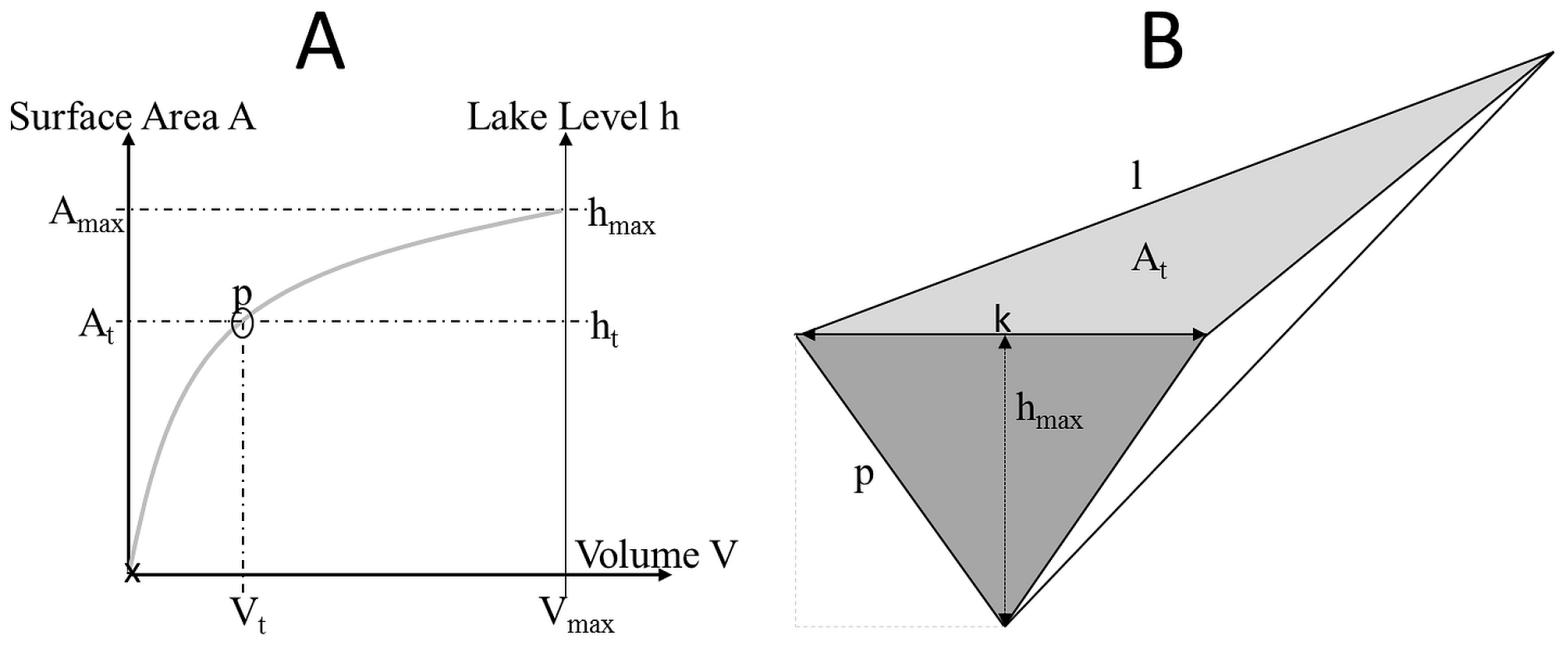
Reservoir basin shapes determine the extent of water level fluctuations. **A:** Relationship between the surface area *A*, lake level *h* and volume *V* of a reservoir optimized from [21] where the surface area and the lake level decreases with the volume. *V_max_* is the maximum lake volume, *A_max_* the maximum surface area and *h_max_* the maximum lake level. *V_t_* is the volume at time *t*, *h_t_* the lake level at time t and *A_t_* the surface area at time t. The grey line depicts the outline of a reservoir basin cut in half and rotated 90 degrees around the x-axis (with the X being the deepest point at the center of the basin). The black circle indicates the value that the shape parameter p assumes (slope at any given level of the reservoir basin). **B:** simplified model for the assumed reservoir morphometry in our case study: a top down pyramid that is diagonally cut (*A_t_*  =  surface Area, l  =  length, p  =  shape of the shoreline, k  =  width of the dam, and the dark grey area  =  dam),


To be able to translate the changes in the storage volume into WLF, we use a generic mathematical relationship between the storage volume and the water depth above the deepest part of the lake, which we term the depth-volume relationship [20] (Fig. 2 A). This relationship depends on the depth, the volume, and the slope of the reservoir basin [21] and is given for a dammed river by

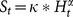


where *S_t_* is the stored volume level of the lake at time *t* received from the storage balance equation, *H_t_* the water depth of the lake at *t* and *κ* a constant based on the relationship between depth and volume. *κ* depends on the reservoir basin slope (Fig. 2 A, B). The more open and flatter the valley, the larger is *κ* [22], [23]. The value *α* defines the concavity of the basin (for further details see [20], [23]).

The WLF can then be determined via the depth change between period *t* and *t+1.* The mathematical equation for the extent of the *WLF* is given by


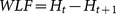


The estimated value for *WLF* represents the water level change between the depth *H_t_* of period *t* and the depth *H_t+1_* of the period after it, related to the hydropower production process.

### Specific model, definition of exogenous parameters: Lake Goeschener Alp Case Study

To verify the applicability of our model, we test it in a case study. As model case we choose Lake Goeschener Alp (henceforth Lake GA), a large-scale hydropower storage plant reservoir in central Switzerland without any pumping system (dam wall height ca. 100 m, max. vol. approx. 75 million m^3^, capacity ≥ 40 MW) [24]. For illustration purposes, we present a Fig. in the supplementary material with a list of all reservoirs ranked by reservoir volume including Lake GA (S1 Fig.). The reservoir is fed by run-off from the 95 km^2^ wide catchment, which comprises a glacier (the Dammaglacier, covering 20% of the total catchment area) [25]. In larger reservoirs (> 10 MW capacity) the variation in head throughout the planning time horizon is so small, that it does not significantly affect any operating decisions [26], [27]. In short-term studies, the head effects are usually neglected altogether [27], [28]. As we model a large reservoir on mid-term time scales here, we obtain the head *H_t_* as linear function of the average storage level at beginning *t* and the end of the time *t-1*.

The real WLF data from the reservoir were made available to us (for research purposes only) by the Kraftwerk Göschenen AG, Remo Infanger, Postfach, CH-6002 Luzern, Switzerland, which might be contacted for future permissions. To test the ability of our model approach to capture real-world WLF and to provide an assessment tool for potential changes in the market and natural environment, we will apply the following approach: firstly, we simulate a historic WLF benchmark case (Sc1) and compare it to the observed values (i.e. real water levels). Secondly, we carry out scenario estimates with altered inflow (Sc2) and energy price (Sc3) values. Finally, we will provide the model inputs for the benchmark and scenario cases. The results are provided in the next section.

#### Specific model input data

The length of time *t* and the total time horizon *T (t  =  [1…T])* can vary based on available data and the desired detail of output resolution. For our assessment, we capture the time horizon of the hydrological year 2010–2011 at all days *t*, but consider energy generated only during peak load hours in the time period *T*. As definition of peak load hours we apply the EPEX definition [29], which describes peak loads as hours from 09:00 a.m. to 20:00 p.m. During this period, electrical power is in higher-than-average demand, because e.g. consumers use their stoves for cooking [30].

We use energy prices of 2010–2011 from the European Energy Exchange [29]. Prices are the Swissix spot market day peak prices of the period October 2010 to September 2011.

**Figure 3:**
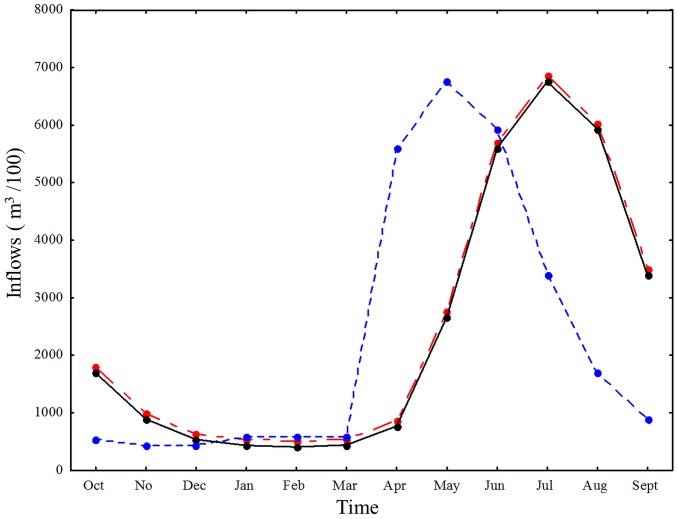
Reservoir inflows change with scenarios. Seasonal development of inflows (m^3^ month^−1^) based on the present-day data as used for the hydro-economic model (solid line: reference scenario (Sc1)) and based on the climate change scenario (Sc2, blue dotted lines) that predicts a change in run-off pattern due to increased glacier melt in the future, and based on the price change scenario (Sc3, red dashed line) that predicts an increase in turbine capacity and price volatility. Note that scenarios Sc1 and Sc3 assume identical inflows and the lines and points here are merely jittered by 1000 m^3^ to improve visibility.

For hydrological input values, we calculate the inflow into the case study reservoir (see below) based on specifically compiled databases for estimation of water balances in catchments of the Swiss Alps (see [31], [32] for details on the databases). The catchment area was defined in ArcGIS, based on the raster cell dataset, and combined with the database's information on the average (of the period 1981–2000) water balances (run-off, precipitation, etc.) of catchments in Switzerland [32]. By combining monthly mean run-off values in m^3^ s^−1^ for each raster cell in the catchment area [33], we could calculate the mean monthly inflow into Lake GA (Fig. 3). Therefore, inflow captures the run-off from the water catchment area of the storage lake, as well as direct inflows from running waters.

### Modelling water levels from the Lake Goeschener Alp and comparing them to other alpine reservoirs

In theory, the relationship between reservoir shape and volume (Fig. 2) can also be used to assess the relationship between water level and storage volume of the reservoir. By definition, in our generic model, we cannot make such specific assumptions concerning shape and volume relationships. For this specific case study, however, we assume a specific reservoir shape to be able to translate storage volume in m^3^ into water levels in m. We would like to stress that other morphometries (i.e. slopes of basins) can be specifically addressed, assuming a different relationship (see Liebe et al. [20] for worked examples on other types of reservoir morphometries). For details on how the values of *κ* and *α* are determined, please refer to [20], [22], [23]. For ease of comparison of Lake GA water levels with other lakes, we expressed Lake GA water levels as % of total storage capacity, whereby the minimum storage capacity is the level of water at maximum drawdown. Below that level, there is still water in the reservoir, but this water is needed for dam stabilization.

In practice, to assess the comparability of Lake GA water levels with other reservoirs, and to test whether our model data had a sufficient across-the-market applicability, we compiled water level data from 85 alpine reservoirs in the Swiss Alps. The data was obtained from the database of the Swiss Federal Office of Energy [34] which records the storage levels of all Swiss reservoirs every Sunday at midnight (however, only as % of total storage capacity). See S1 Fig. for a list of all the reservoirs. The data are freely available online, and we manually extracted the storage data for the hydrological year 2010–2011 from the database.

### First model application: altered WLF due to climate change (climate change scenario Sc2)

The future development of hydropower will be fastest in mountainous areas where valleys exist that can easily be dammed to create reservoirs [15]. To test whether our model can capture future developments of WLF due to climate change, we used existing climate change forecasts as exogenous hydrological parameters. The run-off and hence inflow into reservoirs in such mountainous areas, can be strongly influenced by the seasonality of snowfall and glacier melt. This seasonality will likely respond to climate change [15], [33], [35]. Confirming this notion, a recently published study outlined how climate change in the period 2021–2050 will affect the run-offs in the very catchment, where our case study reservoir is located [25], [35], [36]. The forecast, which we used as exogenous parameters for our model, was based on climate-models of the EU-project ENSEMBLES [37]. ENSEMBLES uses the IPCC scenario ‘moderate warming’ and proceeds to a general circulation model. A regional climate model is applied for dynamical downscaling and a hydrological station-specific climate change scenario is implemented for statistical downscaling. Finally, the downscaled climate scenarios were combined with an existing detailed hydrological model. This specific hydrological model was based on a large-scale and 3-year-long empirical data sampling of the complete reservoir catchment of Lake GA. The resulting combined model PREVAH directly generated the changed distribution of inflows that we used for the climate change scenario Sc2 (for further details on the climate scenario calculation see www.c2sm.ethz.ch/services/CH2011; and www.cces.ethz.ch/projects/clench/BigLink, and the references [15], [33], [35]) The rationale for this inflow change rests upon an accelerated glacier melt of the Dammaglacier due to warmer temperatures [33]. Following this predicted earlier onset of the glacier melt, the period of maximum inflow in the summer moves forward by three weeks [33] (Sc2, Fig. 3). The magnitude of the inflows changes only marginally: the maximum inflow increases by 0.6% [33]. In short: the existing Lake GA case study specification of the generic model was further modified by changing the Lake-GA-specific hydrological input values.

### Second model application: altered WLF due to change in energy prices (price change scenario Sc3)

Any change in the operating schedule of a hydropower plant only makes sense in light of the price of electricity. The influence of climate change on water balances and hence WLF in reservoirs are hard to predict, just as much as market-effects, that change the operating schedule and hence WLF in the reservoir.

For instance, one plausible scenario for the future of energy markets worldwide is a growing development of variable renewable energies: solar and wind-power. Both sun and wind will produce energy following diurnal solar and wind patterns. Hence, like the weather, electricity peak and off-peak prices in the future will be stochastically distributed over time.

Operators of hydropower reservoirs can meet this future increase in volatility of prices by extending the capacity of their turbines [38]. This will enable them to more readily respond to price peaks by discharging more water in a shorter time (more m^3^ s^−1^) [38]. In Lake GA, for instance, the operator could increase the capacity by upgrading from four to six turbines with a capacity of 7.5 m^3^ s^−1^ each. Therefore, we tested whether our method allows for modelling the development of WLF when assuming changing energy markets and an operator's response to these changing energy markets in form of an increase in maximum turbine capacity. To simulate a change in the price distribution to which the operator will respond, we assume a higher volatility of energy prices [39]. We base our simulation on a scenario of the Swiss Federal Office of Energy [39] which accounts for changes in the demand and supply-side conditions due to future development of variable renewable energies. The scenario predicts a 17.25% increase of daily peak prices by the year 2035 [39] compared to the mean peak electricity price of 2011. We incorporate this prediction into our model by manipulating the highest 20% of the energy peak prices with an increase by 17.25%. Because the scenario also predicts an increase in price volatility, we accordingly manipulated the lowest 20% of the peak prices by decreasing them by 17.25%.

### Statistical analyses for model evaluation and comparison of scenarios

To assess the match between the modelled and the real data, and to compare the observed lake level data with the lake level from the model (reference scenario Sc1), we calculated the Nash-Sutcliffe criterion. The Nash-Sutcliffe criterion or model efficiency coefficient is used to assess the predictive power of hydrological models. The Nash-Sutcliffe coefficients can range from −∞ to 1, whereby 1 corresponds to a perfect match of modeled and observed data. An efficiency less than zero occurs when the mean of the observed data is a better predictor than the model data. The closer the values approach 1, the more accurate is the model.

**Figure 4:**
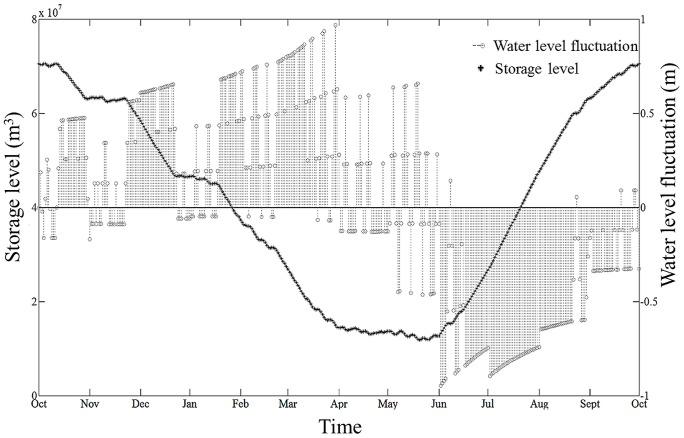
Storage volume and water level fluctuations change across seasons. Seasonal development of the modelled storage level (volume of water in the reservoir in m^3^ (solid curve) and according daily WLF (grey dotted columns)) in Lake GA in 2010–2011. Lower levels in March through June indicate a lower storage volume in the reservoir, from which the water level increases towards October until it decreases again. WLF are depicted as the daily maximum or minimum water level change (in m) in relation to the previous day, i.e., if the column is above the zero isocline of the right y-axis, the WLF represents an increase in water level relative to the level found the prior day. Accordingly, if columns point below the zero isocline, they constitute a decrease in water level. Please note the different resolutions of the y-axes.

Because the Nash-Sutcliffe criterion is sensitive towards outliers, we first screened the data for outliers using the Grubb's test for outliers [40]. As mentioned above, *WLF* represent the water level change between the depth *H_t_* of period *t* and the depth *H_t+1_*. Expression of WLF as the absolute differences in water level between two days in meters resulted in daily values of level-change differing in their algebraic signs. This change of algebraic sign describes a relative in- or decrease in water level, regardless of the amplitude (Fig. 4). We therefore chose to express WLF in the form of changing levels relative to the total capacity of the reservoir. Daily WLF were thus described as percentage change relative to the reference state of a full reservoir which corresponds to 100%. This presentation avoided sensitivity of the Nash-Sutcliffe criterion [41] and allowed for a comparison with relevant reference data from other reservoirs, which were available in the same format. However, for coherence to our case study, and to illustrate the generic and specific model output, we additionally present the daily WLF data as difference in m in the supplementary materials (S2 and S3 Figs.).

To test for differences in amplitudes of WLF across scenarios, we used the Levene's test for homogeneity of variance. The Levene's test indicates whether variances across different groups are from the same sample population. We assumed the variances of the scenarios (Sc1, Sc2, Sc3) to capture the amplitudes because the variance describes how far a set of values is spread from the mean. If the Levene's test is significant, then this suggests that the way in which lake levels are spread around the mean is different across scenarios. A significant Levene's test also confirms heteroscedasticity in the data. Hence, to specifically test how the course of the WLF changes across the season in different scenarios, we conducted a test for homogeneity of slopes across scenarios. In contrast to traditional ANCOVA models, the homogeneity of slopes design does not assume variance homogeneity across factors. It rather tests whether continuous predictors (days in our case) have different effects (variation of WLF) at different levels of categorical predictors (scenarios Sc1, Sc2, Sc3 in our case) [40]. This allows us to test whether seasonality is different between different scenarios by comparing the slope of the relationship between time (used as a covariate) and water level in each scenario. This test could also be used to test for the difference in the seasonality of WLF in Lake GA and other alpine reservoirs.

## Results

### The hydro-economic model sufficiently describes both the storage level curve and the WLF of the case study Lake GA (Sc1)

**Figure 5:**
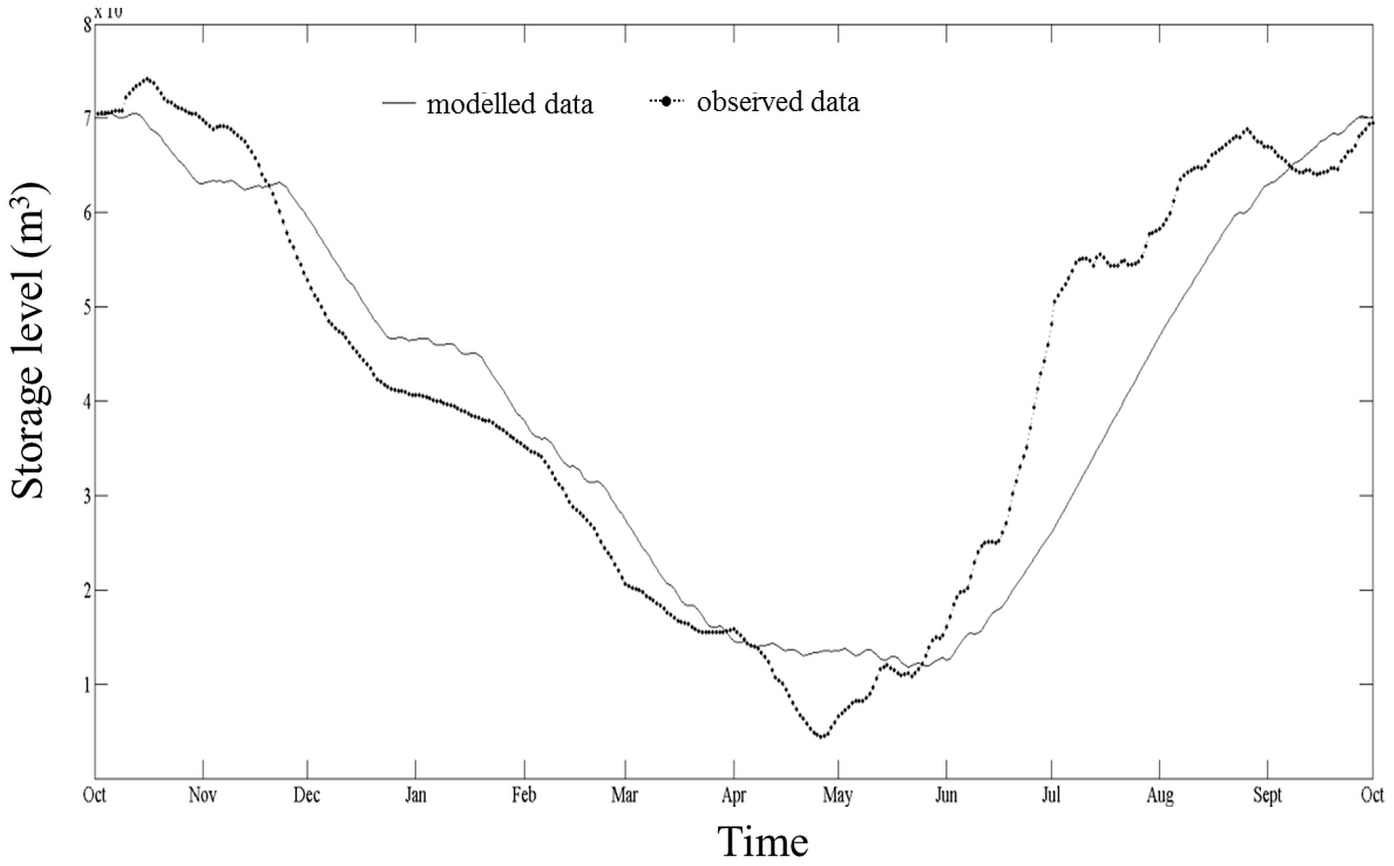
Modelled storage levels match observed storage levels. Seasonal development of storage level (m^3^) (volume of water stored) based on daily values in Lake GA. Solid curve: modelled daily values (reference scenario Sc1), dotted curve: real storage level data provided as daily water levels by the power plant operator Lake GA for 2010–2011.

The main output of the optimization function (equation I) was the generation of a reservoir storage level curve specifically for Lake GA (Fig. 4). The storage level curve shows how the reservoir level changes during the time horizon *T*. The progression of the storage level curve follows the water balance of the reservoir, as calculated from the water balance equation (III). The water storage decreases over seven months until, eventually, reaching a minimum in spring. From this minimum, the storage volume increases within four summer months, reaching the maximum storage volume again in autumn. From the progression of the storage curve, the values of each reservoir level change are used as input *S_t_* for each day *t* for the simulation of the depth-volume relationship. Here, we obtain the WLF which result in WLF relative to the prior day (Fig. 4). WLF predominantly occur as a relative decrease, i.e., loss of littoral zone due to shoreline displacement until spring. From then on, the WLF occur as a relative increase, i.e., inundation, of littoral zone. It should be noted that WLF frequently change direction. This means that water levels might decrease or increase relative to the prior day, regardless of whether they occur in a period of general emptying or filling of the reservoir (Fig. 4). The highest amplitude of WLF can be observed in spring and early summer months. The maximum negative volume change (maximum storage level minus minimum storage level) in the period is 60,656,100 m^3^, which corresponds to nearly a 65 m lake level change in eight months. The comparison of the modelled storage curve and the real data revealed a good match (E_Nash-Sutcliffe_  =  0.867, Fig. 5). The Grubb's outlier test did not find any outliers in the data (Grubb's Test Statistic _modelled data_  = 1.42, p = 1.0; Grubb's Test Statistic _real data_ = 1.69, p = 1.0).

**Figure 6:**
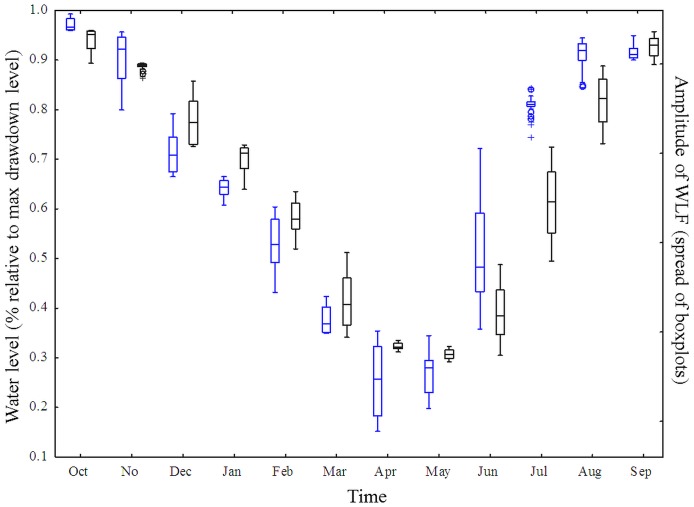
Modelled water level fluctuations match observed water level fluctuations. Seasonal development of WLF in Lake GA in 2010–2011 based on the storage levels calculated by the hydro-economic model (black boxplots: simulated data, reference scenario Sc1) and based on the real data on storage levels provided by the operator (blue boxplots: observed data). The amplitude of the WLF is depicted as the inner and outer spread of the boxplots.

The close match between modelled and observed storage levels is further illustrated by a similar distribution of the monthly means of modelled and observed WLF (Fig. 6). There were some differences in the amplitudes: the reference scenario seemed to underestimate the amplitude in spring and early summer but overestimate the amplitudes in the winter month. However, there was an overall good fit of modelled and observed data (E_Nash-Sutcliffe_  =  0.793). Here, too, the Grubb's test for outliers did not identify any outliers (Grubb's Test Statistic _modelled data_  = 1.51, p = 1.0; Grubb's Test Statistic _real data_ = 1.97, p = 1.0).

### The seasonal water levels in the case study Lake GA are similar to those of other alpine reservoirs

**Figure 7:**
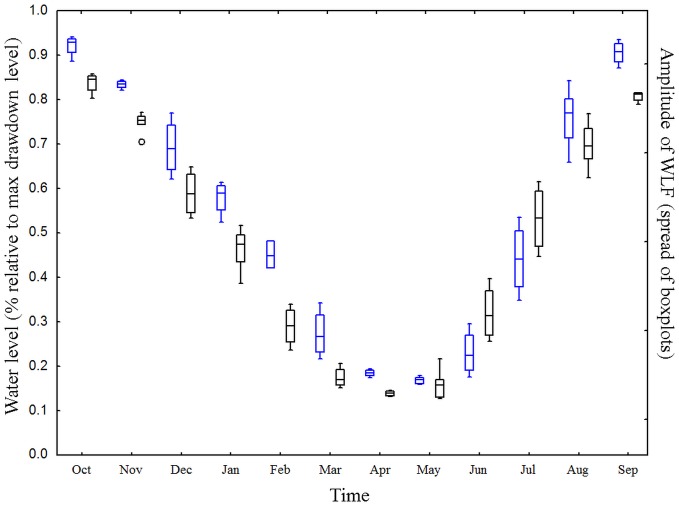
The modelled seasonal changes in water levels are similar to those in other alpine reservoirs. Seasonal development of WLF in Lake GA based on the mean storage levels of all Swiss reservoirs (black boxplots) compared to the WLF calculated by the hydro-economic model (simulated data, reference scenario Sc1, blue boxplots). For a list of all the included reservoirs please refer to S1 Fig. For more information on the data, see the Materials and Methods section.

The seasonal progression of the water levels of Lake GA showed a high similarity with the mean storage level of 85 other alpine hydropower reservoirs (Fig. 7). The compiled Swiss data did not differ significantly from the model data (homogeneity of slopes test, F _2,1092_ =  0.17, Sum of Squares = 13.0, p = 0.89). In general, both Lake GA and the other alpine reservoirs show a decreasing water level until early spring, from when on water levels rise again. Also, the amplitude of WLF appears similar in Lake GA as well as other alpine reservoirs: both data show an increased amplitude of WLF in early summer (especially June and July, Fig. 7)

### Climate change (Sc2) and price change (Sc3) scenarios lead to different patterns of WLF

**Figure 8:**
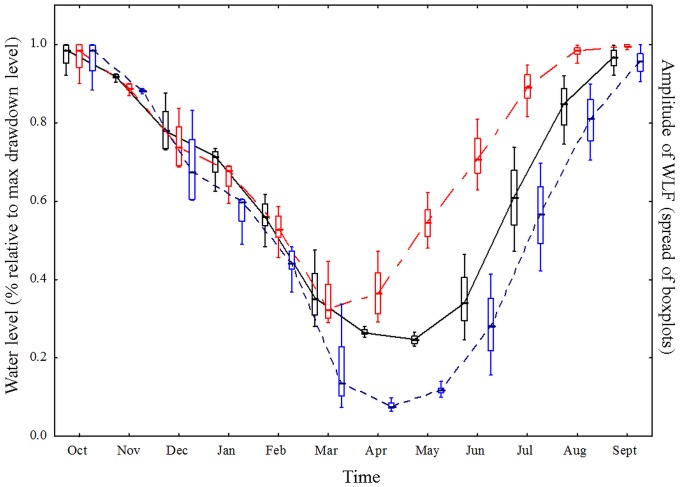
Water level fluctuations differ across price and climate change scenarios. Monthly WLF in Lake GA as calculated by our hydro-economic model (Sc1 black, solid line) and as calculated for two different scenarios (Sc2 red boxplots and red dashed line): change in the seasonality of run-off from the lake's catchment due to glacier melting as predicted by a climate change model specifically designed for Lake GA. Sc3 (blue box plots, and blue dashed line): change in the capacity of the Lake GA power plant in the form of an increased turbine capacity as expected for future development of hydropower in the greater catchment area where Lake GA is situated. For better visualization, the means are connected by lines. Note that WLF are average fluctuations with the first of the month's water level as the starting level and that WLF, although presented separately here, accumulate over the season.

The patterns of WLF assessed by our model (Sc1) responded to further modifications of the hydrological and economic input values. The significant Levene's test indicated that variances across scenarios were different (Levene's F_2,1095_ = 42.97, Mean Sums of Squares 0.92, p<0.001). Following a predicted earlier onset of the glacier melt in the climate change scenario (Sc2), the period of maximum inflow in the summer moved forward by three weeks [35] (Fig. 3). The magnitude of the inflows changed only marginally with the maximum inflow increasing by merely 0.6% [35]. The modelled development of WLF under this specific climate change scenario shows that WLF with larger amplitude occur earlier in the hydrological year and WLF were less pronounced in March and April (Fig. 8, S3 Fig.). This results from an earlier filling of the reservoir due to increased inflows from glacier melt, which provides more water for turbination at an earlier point in time (Fig. 3).

The amplitudes of the climate change (Sc2) and the reference scenario (Sc1) are similar (Coefficient of variance Sc1 = 0.27, Sc2 = 0.24) but show differences in their seasonal distribution. While Sc1 and Sc2 show a similar development until early spring, the WLF in Sc2 occur with lower amplitudes until the end of the year. Due to the fact that the first negative range of Sc1 is in spring, the amplitudes shifted to the end of the summer (Fig. 8).

The most striking difference across scenarios is the higher variance (i.e. amplitude in WLF) in the price change scenario Sc3 compared to Sc1 (Coefficient of variance Sc3 = 0.39, Sc1 = 0.27, Fig. 8), especially in winter and spring. The Sc3 scenario also shows the highest maximum of WLF. This reflects a higher discharge through the turbines when the modelled prices are higher than in other scenarios. Overall, the price change scenario reflects the increase in discharge with higher prices as ultimate and higher turbine capacity as proximate factors.

The results of the test for homogeneity of slopes confirm that the development of WLF across seasons differs significantly between scenarios (F =  3.53, Sum of Squares = 0.649, p = 0.030). The significant interaction term scenario x days further demonstrates that the type of scenario influences the seasonality of WLF, i.e., how daily WLF are distributed across the year (F =  25.61, Sum of Squares = 2.33, p<0.001).

## Discussion

### Model accuracy and uncertainty factors

We acknowledge that many other factors than water withdrawal for turbines can affect the water level and WLF in lakes. Evaporation and other variables of the water balance equation, the water exchange with soils, and seepage of water at the lake bottom can also influence the water balance of a lake and hence cause WLF [42]. Recent advances in hydroinformatics actually allowed for an extremely accurate modelling of water levels [16]. However, this accuracy was only achieved in lakes, whose water level is solely governed by natural hydrological processes [16]. Hydrological processes are likely to play only a minor role in reservoirs, at least in determining the amplitude of WLF. This becomes obvious from comparing the amplitudes of natural and anthropogenic WLF (Table 1, [12]). Studies of WLF in other systems confirm the notion that the operation of the reservoir for hydropower production will override other hydrological factors in determining WLF [5], [42], [43].

Clearly, by combining two disciplines, hydrology and economics, we were forced to simplify the assumptions at both ends. For hydrological data this means that the inflow calculation is based on modelled values which reflect the mean inflows into Lake GA during the period 1981–2000 rather than time-matched observations parallel to the model's time horizon. The actual run-offs in the individual year can differ from this long-term average. The comparison between the model results and the operator's data (Fig. 3) therefore shows that the dataset's inflow values lead to a deviation in the modelled storage volume level curve, compared to the real data. Furthermore, evaporation from the lake surface, infiltration from the lake bed, and precipitation directly into the lake (not as run-off from the surrounding catchment) are not considered in our model. This means, depending on which climate change or price change forecast one applies, the resulting scenarios of the hydro-economic model will inherit the input model's degrees of uncertainty. This would result in an additionally higher uncertainty of the WLF scenarios [33].

### Possible extensions of the model through different exogenous parameters

Our generic model can be extended and tailored to other specific case studies by incorporating more catchment-specific hydrological models, more elaborate models of price development, and more detailed climate change models. The necessary simplification of the hydrological parameters can be alleviated by combining our generic model with advanced hydroinformatic models [16]. This will provide more detailed and more locally specific information on, e.g., inflows and evaporation*i_t_*. Apart from hydrological parameters, our model can also be extended with a stochastic modelling of energy prices *p_t_*. The operation scheme of the power plant varies with energy prices. Our application of a generic deterministic model cannot fully account for such variation. Energy prices are typically uncertain and respond to a multitude of factors. Advanced stochastic models for dealing with the uncertainty in energy price development are available and might be incorporated into the hydro-economic model. In the supporting information, we provide more details and references on how exactly our model can be extended (S1 File). At any case, further model extensions require high computing capacity and vary starkly depending on the assumed developments [44]. More detailed modifications in exogenous parameters will inevitably result in an increased case-specificity of the model. Because we aimed for a presentation of a generic model and provide a case study for illustration purposes, we deemed more exemplary modifications of parameters as beyond the scope of our study. However, such a combination is absolutely possible if specific climate or price change scenarios are at hand or developed for a specific study system. This will then allow an estimation of the development of WLF, depending both on environmental forces, such as climate change, and on the development of prices. Future research into this area can proceed from our basic model. It should also be stressed that the climate change models and price change models were based on published literature specifically dealing with our case study. Exogenous parameters from other site-specific studies are frequently available and can be used to tailor the model to a variety of further case studies.

### Translating WLF into shoreline displacement or inundation – the importance of reservoir basin shape

The seasonal change in shoreline displacement can be easily assessed using our model by calculating the area that falls dry, i.e., beaching, or is inundated in relation to WLF.

The calculation of the extent of shoreline displacement or inundation strongly depends on the reservoir basin shape. The basin slope determines the slope of the littoral and thus the magnitude of shoreline displacement per unit water level change. In this study, a simplified shape of a diagonally cut pyramid was chosen as the best match for our case study.

This remains a simplifying assumption that does not precisely reflect the shape of the reservoir Lake GA. Despite this variation in shape among reservoirs, the calculation we use to estimate the relationship between reservoir level and storage volume, sufficiently describes most alpine reservoirs [20]. This assumption is supported by the similarity between the storage level curve of Lake GA and the mean of 85 Swiss reservoirs combined. The increase in amplitude of WLF towards the season in which the lake is less full, can at least partly be attributed to the reservoir's shape. If there is less water available within the convex slope of the reservoir, then discharged volumes of water will correspond to higher changes in water level compared to a full reservoir. This phenomenon highlights the importance of more detailed information on the reservoir morphometry for future empirical research on WLF. The match between real and modelled shoreline displacement will become more accurate when assuming a more detailed reservoir basin shape. Hence, detailed bathymetric maps will greatly improve the accuracy of our predictions of the effect of WLF on shoreline displacement. This will also further improve the match between modelled and observed WLF.

### Ecological relevance of WLF amplitude and seasonality

The responsiveness of aquatic biota – from primary producers to fish – to changes in WLF epitomizes the importance of understanding and managing WLF. The seasonality of WLF resulting in freshly beached or inundated littoral area is an important trigger for the life cycles of many aquatic and semi-aquatic species. The assessment of our hydro-economic model shows changes in both the amplitude and the seasonality of WLF, triggered by two relevant future developments: climate change and changes in the energy market. Especially the significant difference in the homogeneity of slopes indicates a divergence in WLF across the season among scenarios. This is highly relevant because researchers will be interested in the variation of shoreline displacement throughout the season, since this will allow to better assess the ecological effects of WLF. Based on the comparison of Lake GA and other reservoir data, the increase in the amplitude of WLF within the early summer months appears to be a general pattern in alpine reservoirs. This has ramifications for assessing and managing the ecological effects of WLF. Changes in the amplitude and seasonality of WLF will affect species' phenologies that frequently match natural cycles in water levels. If high amplitudes of WLF fall into a period in which biota or biological processes are particularly sensitive, then this period requires special attention from researchers and managers. For instance, controlled peaks in water levels can instigate the spawning and support populations of littoral spawning fish [45], [46]. Conversely, littoral invertebrates are sensitive to lower water levels in winter: during winter, many species rely on specific habitats that can be lost if WLF expose the littoral zone to temperatures below zero degrees Celsius [45].

Our model results of the climate change scenario suggest that alterations in run-off and glacier melt, as they are predicted from a variety of climate change scenarios, can affect the seasonality of WLF. Lakes in general are sensitive to climate change because seasonal nutrient cycles and phenologies of aquatic biota are tightly matched [7], [45]. Moreover, mis-matches of species phenologies due to changing seasonality of WLF are likely to impair the ecosystem functioning of lakes [47], [48], [49]. For example, reed stands (*Phragmites australis*) on lake shorelines provide important ecosystem services, such as pollutant retention, but are in sharp decline across many ecosystems [50]. The effect of extreme water levels is believed to be a major stressor responsible for this decline. Such WLF are further predicted to negatively act in concert with higher surface-water temperatures: extreme water levels and higher temperatures both foster the growth of parasitic reed fungi which can cause reed die-off [51], [52].

Even more drastic changes to WLF, at least in terms of higher amplitudes, can be expected from the development of volatile renewable energies and accompanying alterations in energy policies. It remains unstudied how and if changes in the seasonality and amplitude of WLF translate into more profound ecological effects. Our results, however, suggest that the outcome of climate change and the way in which the development of hydropower is progressed, can lead to future changes in WLF.

## Conclusions

A sustainable development of hydropower, drinking water production, and inundation calls for a compromise between maximization of energy production and preservation of aquatic life, with all its values for society. Much progress has been made in establishing environmental flows in the form of artificial discharges, which mimick or approximate natural flows (*in sensu* [53], reviewed in [54]). For progress towards a sustainable development of hydropower, its benefits have to be weighed against possible detrimental effects. This applies to both anthropogenic flow regimes and anthropogenic WLF. Such a cost-benefit analysis has to be informed by solid empirical evidence. Evidence on which flows can be implemented to maximize both the ecological integrity of pristine flows and the produced energy is mounting for lotic systems downstream of reservoirs (reviewed in [54]). To our knowledge, however, there are no data on how such a compromise can be implemented for the water levels within the reservoir above the dam. Our hydro-economic model presents a fundamental method to start addressing such questions. We posit that applied ecosystems ecology can inform about specific requirements of ‘environmental water levels’. Based on our hydro-economic model, it can then be calculated how such ‘environmental water levels’ can be approximated. This will facilitate a compromise between the conservation of water levels relevant to the ecosystem and the maximization of energy production. For example, controlled seasonal beaching or inundation of the shoreline can create temporary spawning or nesting grounds for fish or birds [10], [55]. This will make water temporarily unavailable for discharge through the turbines. In analogy to the provision of certain flows in the case of environmental flows, by the help of our model, research can be instigated that allows for minimizing such losses of water for the operator while approximating environmental water levels. Such a methodology will ultimately advance our understanding of how an efficient management can maximize both economic revenue and ecological integrity of water resources.

## Supporting Information

S1 Fig **Alpine reservoirs.** List of reservoirs in Switzerland, ranked by their capacity. The reservoir Lake Goeschener Alp (in the manuscript referred to as Lake GA) is indicated with a blank column and the mean volume of lakes is indicated with a grey dashed column.
(TIF)

S2 Fig **Modelled water level fluctuations match observed water level fluctuations.** Seasonal development of WLF in m relative to the prior day in Lake GA in 2010/11 based on the observed storage levels (black boxplots) and the modelled from the hydro-economic model, reference scenario Sc1). Medians are connected by solid black and dashed blue lines for observed and modelled data, respectively.
(TIF)

S3 Fig **Water level fluctuations change with different climate change and price change scenarios.** Monthly WLF in Lake GA as calculated by our hydro-economic model (Sc1 black boxplots and black, solid line) and as calculated for two different scenarios: Sc2 (red boxplot and red dashed line): change in the seasonality of run-off from the lake's catchment due to glacier melting as predicted by a climate change model specifically designed for Lake GA. Sc3 (blue boxplots and blue dashed line): change in the capacity of the Lake GA power plant in the form of an increased turbine capacity as expected for future development of hydropower in the greater catchment area where Lake GA is situated. For better visualizations the means are connected by lines and outliers are removed. Note that WLF are averaged fluctuations with the first of the month's water level as the starting level.
(TIF)

S1 File **Details on possible extensions of exogenous parameters of the hydro-economic model.**
(PDF)

S2 File **Raw data table.**
(PDF)
