# Supplementary figures and images for: A Hydro-Economic Model for Water Level Fluctuations: Combining Limnology with Economics for Sustainable Development of Hydropower
Source: PLoS One. 2014 Dec 19;9(12):e114889. doi: 10.1371/journal.pone.0114889 (PMC4272295; doi:10.1371/journal.pone.0114889)

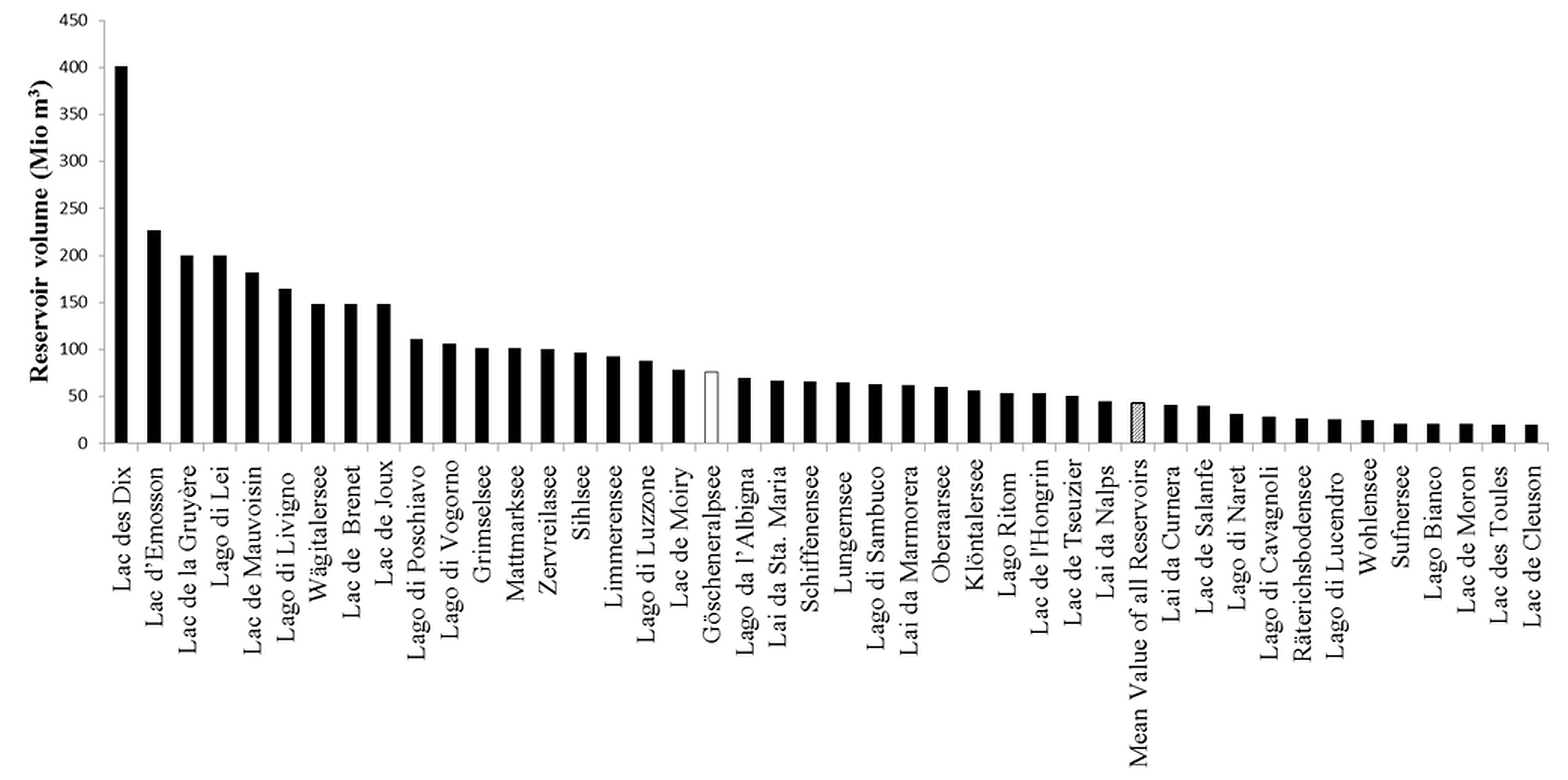

Supplement: S1 Fig — Alpine reservoirs. List of reservoirs in Switzerland, ranked by their capacity. The reservoir Lake Goeschener Alp (in the manuscript referred to as Lake GA) is indicated with a blank column and the mean volume of lakes is indicated with a grey dashed column. (TIF) [file pone.0114889.s001.tif]

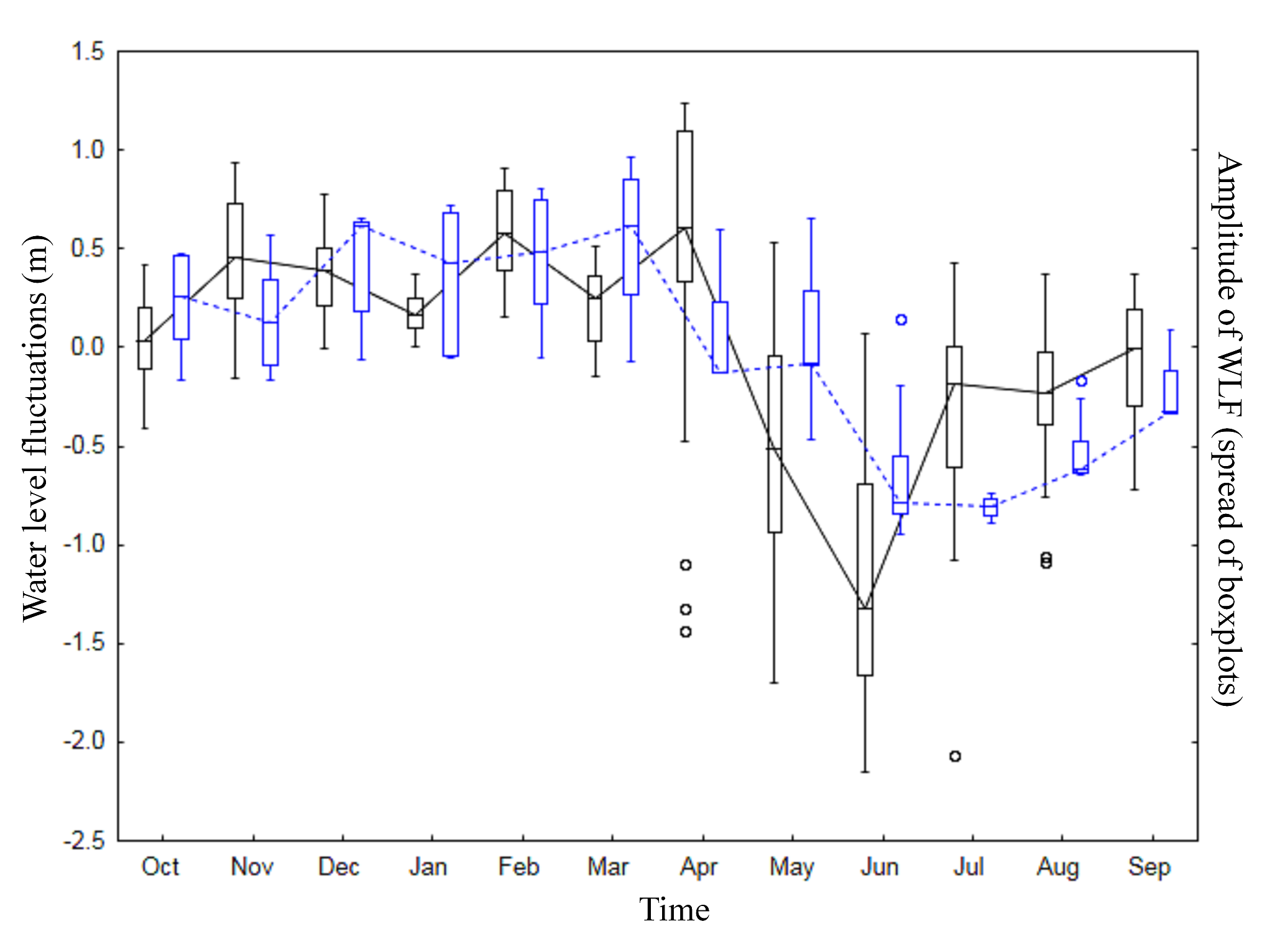

Supplement: S2 Fig — Modelled water level fluctuations match observed water level fluctuations. Seasonal development of WLF in m relative to the prior day in Lake GA in 2010/11 based on the observed storage levels (black boxplots) and the modelled from the hydro-economic model, reference scenario Sc1). Medians are connected by solid black and dashed blue lines for observed and modelled data, respectively. (TIF) [file pone.0114889.s002.tif]

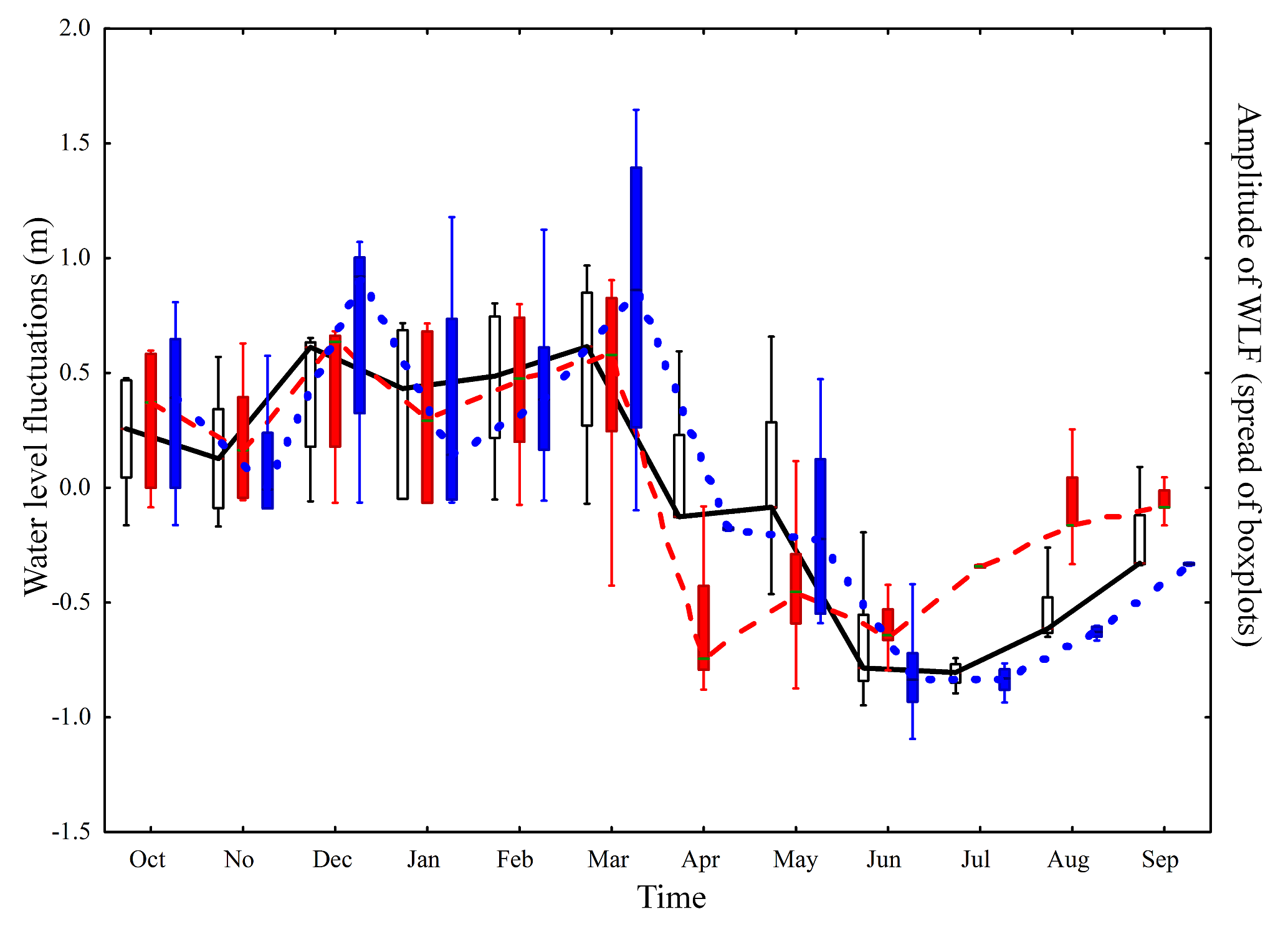

Supplement: S3 Fig — Water level fluctuations change with different climate change and price change scenarios. Monthly WLF in Lake GA as calculated by our hydro-economic model (Sc1 black boxplots and black, solid line) and as calculated for two different scenarios: Sc2 (red boxplot and red dashed line): change in the seasonality of run-off from the lake's catchment due to glacier melting as predicted by a climate change model specifically designed for Lake GA. Sc3 (blue boxplots and blue dashed line): change in the capacity of the Lake GA power plant in the form of an increased turbine capacity as expected for future development of hydropower in the greater catchment area where Lake GA is situated. For better visualizations the means are connected by lines and outliers are removed. Note that WLF are averaged fluctuations with the first of the month's water level as the starting level. (TIF) [file pone.0114889.s003.tif]
