# Supplementary material for: A Hydro-Economic Model for Water Level Fluctuations: Combining Limnology with Economics for Sustainable Development of Hydropower
Source: PLoS One. 2014 Dec 19;9(12):e114889. doi: 10.1371/journal.pone.0114889 (PMC4272295; doi:10.1371/journal.pone.0114889)
Supplement: S1 File — Details on possible extensions of exogenous parameters of the hydro-economic model. (PDF) [file pone.0114889.s004.pdf]

S1: Details on possible extensions of exogenous parameters the hydro-economic model

Our hydro-economic hydropower model was designed to combine approaches of economic hydropower optimization with hydrological parameters. The main goal was the assessment of water level fluctuations in a hydropower reservoir. The basic premise of our approach is to provide a flexible modular setup that can easily be adjusted to available model capacities. The presented generic model represents the linkage between economic dispatch and WLF. This in turn can be linked to more detailed economic models providing price inputs and market dynamics on the economic side and more detailed hydrological, and climate models on the water inflow side. We would like to emphasize that more detailed programming inevitable makes the model more case-specific because more exact boundaries and exogenous parameters have to be assumed. To pertain to a more general approach in the main manuscript we therefore provide possible model adjustments here in the supplementary materials.

Adjustments to generic model formulation to extend economic aspects

From an ecological perspective the seasonality of WLF is crucial because it affects aquatic organisms' life-cycles (see main manuscript for examples). Based on published literature on hydropower operations, we therefore chose mid-term optimization that shows monthly or weekly resolutions of storage level values for our model (Birger et al. 2001). As a more detailed production optimization, an alternative short-term optimization can be adopted with a time horizon consisting of incremental steps such as hours or quarter-hours. Such a model extension merely requires more detailed price data and potentially higher computation capacities as the model size increases, but the generic formulation remains unchanged (Borghetti et al. 2008; Chang et al. 2001). In the generic model energy prices were assumed as exogenously given. A possible model extension is to approximate real and future prices by stochastic dynamic programming (Eichhorn 2010); i.e., by using time series of historic information to generate price distributions, by forward simulation (a model tries to find possible value developments based on current data sets (Longstaff and Wang 2004)), or by a rolling planning model approach (Troy et al. 2010). Further details how stochastic dynamic programming is used to determine energy prices and inflows could be taken from: Siqueira et (al. 2006) who employed stochastic programming; from Blanco et al. (2001) who modeled prices with geometric Brownian motion; from Birger et al. (2001) who used Stochastic Dynamic programming based on a Markov chain principle; and from Zambelli et al. (2009) who modeled prices and inflows in concert using an open-loop feedback control framework.

Adjustments to exogenous hydrological parameters

The second adjustable aspect of the generic model is its linkage to detailed hydrological inflow and climate models. In the generic formulation this linkage is represented by the external input parameter *natural inflows* i_t. For the climate change scenario S2 we adjusted the base case values according to forecasts specifically modelled for the reservoir which we investigated.

Consequently, the same can be carried out for a large variety of other influences like evaporation depending on the available model capacities. Further details of how climate change in the form of all possible effects on the water-balance of a reservoir catchment can result in different exogenous parameters can e.g. be found in Wang et al. (2014) who used Grey Forecasting Models to forecast precipitation. An overall review of methodologies

developed to capture and forecast the effects of climate change on hydropower reservoirs in alpine regions can be found in Gaudard et al. (2014)

Finally, also the included technical parameters can easily be tailored to the specificities of the investigated hydro plant (Lu et al. 2004). Turbine capacities, minimum and maximum storage levels and further regulatory restrictions can be adjusted and added/omitted following the logic presented in the generic model framework (Schlecht and Weigt 2014).

References

Birger M, Gjelsvik A, Grundt A, Karesen K (2001) Optimisation of hydropower operation in a liberalised market with focus on price modelling. 2001 IEEE Porto Power Tech Proceedings (Cat. No.01EX502):vol.1, 6 pp.

Blanco C, Choi S, Soronow D (2001) Energy Price Processes Used for Derivatives Pricing and Risk Management. Commodities Now

Borghetti A, D'Ambrosio C, Lodi A, Martello S (2008) An MILP approach for short-term hydro scheduling and unit commitment with head-dependent reservoir. IEEE Transactions on Power Systems 23:1115-1124

Chang GW et al. (2001) Experiences with mixed integer linear programming based approaches on short-term hydro scheduling. IEEE Transactions on Power Systems 16:743-749

Eichhorn A (2010) Stochastic Programming in Energy: Theory vs. Practical Application. 6th Annual ÖGOR-IHS-Workshop "Mathematische Ökonomie und Optimierung in der Energiewirtschaft". Institute for Advanced Studies (IHS), Vienna, Austria

Gaudard L, Romerio F, Dalla Valle F, Gorret R, Maran S, Ravazzani G, Stoffel M, Volonterio M. (2014) Climate change impacts on hydropower in the Swiss and Italian Alps. Science of the Total Environment 493:1211-1221

Longstaff FA, Wang AW (2004) Electricity forward prices: A high-frequency empirical analysis. Journal of Finance 59:1877-1900

Lu N, Chow JH, Desrochers AA (2004) Pumped-storage hydro-turbine bidding strategies in a competitive electricity market. IEEE Transactions on Power Systems 19:834-841

Schlecht I, Weigt H (2014) Swissmod-A Model of the Swiss Electricity Market. FoNEW Discussion Paper 2014/01

Siqueira TG, Zambelli M, Cicogna M, Andrade M, Soares S (2006) tochastic Dynamic Programming for Long Term Hydrothermal Scheduling Considering Different Streamflow Models. 9th International Conference on Probabilistic Methods Applied to Power Systems, KTH Stockholm, Sweden

Troy N, Denny E, O'Malley M (2010) Base-load cycling on a system with significant wind penetration. IEEETransactions on Power Systems 25:1088-1097

Wang B, Liang XJ, Zhang H, Wang L, Wei YM (2014) Vulnerability of hydropower generation to climate change in China: Results based on Grey forecasting model. Energy Policy 65:701-707

Zambelli MS, Luna I, Soares S (2009) Long-Term hydropower scheduling based on deterministic nonlinear optimization and annual inflow forecasting models. 2009 IEEE PowerTech Bucharest. pp 1-8
