# Supplementary material for: A Hydro-Economic Model for Water Level Fluctuations: Combining Limnology with Economics for Sustainable Development of Hydropower
Source: PLoS One. 2014 Dec 19;9(12):e114889. doi: 10.1371/journal.pone.0114889 (PMC4272295; doi:10.1371/journal.pone.0114889)
Supplement: S2 File — Raw data table. (PDF) [file pone.0114889.s005.pdf]

S5. Data on inflows and energy prices used in the hydro-economic model

Date [DDMMYYYY]	Energy Prices (S1 & S2) [CHF/Mwh] - Swissix spot market day peak prices	Energy Prices (S3) [CHF/Mwh] - Swissix spot market day peak prices	Date [DDMMYYYY]	Inflows (S1 & S3) [m^3/day]	Inflows (S2) [m^3/day]
01.10.2010	76.45	76.45	01.10.2011	169344	88992
02.10.2010	62.15	51.43	02.10.2011	169344	88992
03.10.2010	52.10	43.12	03.10.2011	169344	88992
04.10.2010	72.71	72.71	04.10.2011	169344	88992
05.10.2010	73.21	73.21	05.10.2011	169344	88992
06.10.2010	72.06	72.06	06.10.2011	169344	88992
07.10.2010	71.22	71.22	07.10.2011	169344	53568
08.10.2010	68.72	68.72	08.10.2011	169344	53568
09.10.2010	67.20	67.20	09.10.2011	169344	53568
10.10.2010	53.63	44.38	10.10.2011	169344	53568
11.10.2010	73.32	73.32	11.10.2011	169344	53568
12.10.2010	80.26	80.26	12.10.2011	169344	53568
13.10.2010	81.49	81.49	13.10.2011	169344	53568
14.10.2010	80.34	80.34	14.10.2011	169344	53568
15.10.2010	82.87	97.17	15.10.2011	169344	53568
16.10.2010	77.58	77.58	16.10.2011	169344	53568
17.10.2010	69.18	69.18	17.10.2011	169344	53568
18.10.2010	91.02	106.72	18.10.2011	169344	53568
19.10.2010	89.98	105.50	19.10.2011	169344	53568
20.10.2010	94.03	110.25	20.10.2011	169344	53568
21.10.2010	102.52	120.20	21.10.2011	169344	53568
22.10.2010	90.00	105.53	22.10.2011	169344	53568
23.10.2010	80.54	80.54	23.10.2011	169344	53568
24.10.2010	79.00	79.00	24.10.2011	169344	53568
25.10.2010	96.08	112.66	25.10.2011	169344	53568
26.10.2010	99.19	116.30	26.10.2011	169344	53568
27.10.2010	92.99	109.03	27.10.2011	169344	53568
28.10.2010	96.46	113.09	28.10.2011	169344	53568
29.10.2010	83.16	97.51	29.10.2011	169344	53568
30.10.2010	62.70	51.88	30.10.2011	169344	53568
31.10.2010	52.37	43.33	31.10.2011	169344	53568
01.11.2010	58.93	48.77	01.11.2011	88992	53568
02.11.2010	73.31	73.31	02.11.2011	88992	53568
03.11.2010	80.36	80.36	03.11.2011	88992	53568
04.11.2010	69.28	69.28	04.11.2011	88992	53568
05.11.2010	81.02	81.02	05.11.2011	88992	53568
06.11.2010	74.99	74.99	06.11.2011	88992	53568
07.11.2010	68.57	68.57	07.11.2011	88992	43200
08.11.2010	83.17	97.52	08.11.2011	88992	43200

09.11.2010	80.54	80.54	09.11.2011	88992	43200
10.11.2010	81.18	81.18	10.11.2011	88992	43200
11.11.2010	79.48	79.48	11.11.2011	88992	43200
12.11.2010	76.45	76.45	12.11.2011	88992	43200
13.11.2010	71.58	71.58	13.11.2011	88992	43200
14.11.2010	46.56	38.53	14.11.2011	88992	43200
15.11.2010	77.39	77.39	15.11.2011	88992	43200
16.11.2010	79.25	79.25	16.11.2011	88992	43200
17.11.2010	76.51	76.51	17.11.2011	88992	43200
18.11.2010	78.88	78.88	18.11.2011	88992	43200
19.11.2010	74.70	74.70	19.11.2011	88992	43200
20.11.2010	70.33	70.33	20.11.2011	88992	43200
21.11.2010	57.82	47.84	21.11.2011	88992	43200
22.11.2010	77.89	77.89	22.11.2011	88992	43200
23.11.2010	81.04	81.04	23.11.2011	88992	43200
24.11.2010	84.80	99.43	24.11.2011	88992	43200
25.11.2010	85.64	100.42	25.11.2011	88992	43200
26.11.2010	86.57	101.50	26.11.2011	88992	43200
27.11.2010	79.24	79.24	27.11.2011	88992	43200
28.11.2010	73.10	73.10	28.11.2011	88992	43200
29.11.2010	88.51	103.78	29.11.2011	88992	43200
30.11.2010	88.39	103.64	30.11.2011	88992	43200
01.12.2010	87.71	102.84	01.12.2011	53568	43200
02.12.2010	95.75	112.26	02.12.2011	53568	43200
03.12.2010	94.80	111.15	03.12.2011	53568	43200
04.12.2010	85.20	99.90	04.12.2011	53568	43200
05.12.2010	81.06	81.06	05.12.2011	53568	43200
06.12.2010	88.85	104.17	06.12.2011	53568	43200
07.12.2010	95.58	112.07	07.12.2011	53568	43200
08.12.2010	89.51	104.95	08.12.2011	53568	40608
09.12.2010	85.36	100.08	09.12.2011	53568	40608
10.12.2010	84.47	99.04	10.12.2011	53568	40608
11.12.2010	75.11	75.11	11.12.2011	53568	40608
12.12.2010	76.62	76.62	12.12.2011	53568	40608
13.12.2010	94.16	110.41	13.12.2011	53568	40608
14.12.2010	99.29	116.42	14.12.2011	53568	40608
15.12.2010	104.82	122.90	15.12.2011	53568	40608
16.12.2010	95.89	112.43	16.12.2011	53568	40608
17.12.2010	94.62	110.94	17.12.2011	53568	40608
18.12.2010	84.96	99.62	18.12.2011	53568	40608
19.12.2010	75.22	75.22	19.12.2011	53568	40608
20.12.2010	83.96	98.45	20.12.2011	53568	40608
21.12.2010	90.11	105.65	21.12.2011	53568	57888
22.12.2010	84.66	99.26	22.12.2011	53568	57888
23.12.2010	81.32	81.32	23.12.2011	53568	57888
24.12.2010	61.16	50.61	24.12.2011	53568	57888
25.12.2010	57.32	47.44	25.12.2011	53568	57888

26.12.2010	74.17	74.17	26.12.2011	53568	57888
27.12.2010	83.24	97.60	27.12.2011	53568	57888
28.12.2010	82.34	82.34	28.12.2011	53568	57888
29.12.2010	75.14	75.14	29.12.2011	53568	57888
30.12.2010	76.15	76.15	30.12.2011	53568	57888
31.12.2010	74.47	74.47	31.12.2011	53568	57888
01.01.2011	42.41	35.09	01.01.2012	43200	57888
02.01.2011	61.97	51.28	02.01.2012	43200	57888
03.01.2011	86.38	101.28	03.01.2012	43200	57888
04.01.2011	81.14	81.14	04.01.2012	43200	57888
05.01.2011	76.79	76.79	05.01.2012	43200	57888
06.01.2011	69.88	69.88	06.01.2012	43200	57888
07.01.2011	72.46	72.46	07.01.2012	43200	57888
08.01.2011	52.69	43.60	08.01.2012	43200	57888
09.01.2011	53.17	44.00	09.01.2012	43200	57888
10.01.2011	83.99	98.48	10.01.2012	43200	57888
11.01.2011	81.72	81.72	11.01.2012	43200	57888
12.01.2011	80.12	80.12	12.01.2012	43200	57888
13.01.2011	79.03	79.03	13.01.2012	43200	57888
14.01.2011	76.09	76.09	14.01.2012	43200	57888
15.01.2011	77.34	77.34	15.01.2012	43200	57888
16.01.2011	64.85	64.85	16.01.2012	43200	57888
17.01.2011	80.62	80.62	17.01.2012	43200	57888
18.01.2011	81.62	81.62	18.01.2012	43200	57888
19.01.2011	79.21	79.21	19.01.2012	43200	57888
20.01.2011	81.12	81.12	20.01.2012	43200	57888
21.01.2011	81.91	81.91	21.01.2012	43200	57888
22.01.2011	81.00	81.00	22.01.2012	43200	57888
23.01.2011	77.00	77.00	23.01.2012	43200	57888
24.01.2011	83.86	98.32	24.01.2012	43200	57888
25.01.2011	81.83	81.83	25.01.2012	43200	57888
26.01.2011	82.30	82.30	26.01.2012	43200	57888
27.01.2011	79.51	79.51	27.01.2012	43200	57888
28.01.2011	79.39	79.39	28.01.2012	43200	57888
29.01.2011	78.64	78.64	29.01.2012	43200	57888
30.01.2011	77.57	77.57	30.01.2012	43200	57888
31.01.2011	84.52	99.10	31.01.2012	43200	57888
01.02.2011	82.72	96.98	01.02.2012	40608	57888
02.02.2011	77.57	77.57	02.02.2012	40608	57888
03.02.2011	77.51	77.51	03.02.2012	40608	57888
04.02.2011	79.52	79.52	04.02.2012	40608	57888
05.02.2011	77.40	77.40	05.02.2012	40608	57888
06.02.2011	74.11	74.11	06.02.2012	40608	57888
07.02.2011	80.69	80.69	07.02.2012	40608	57888
08.02.2011	85.61	100.38	08.02.2012	40608	57888
09.02.2011	84.41	98.97	09.02.2012	40608	57888
10.02.2011	82.55	82.55	10.02.2012	40608	57888

11.02.2011	81.58	81.58	11.02.2012	40608	57888
12.02.2011	77.30	77.30	12.02.2012	40608	57888
13.02.2011	73.02	73.02	13.02.2012	40608	57888
14.02.2011	79.52	79.52	14.02.2012	40608	57888
15.02.2011	80.74	80.74	15.02.2012	40608	57888
16.02.2011	81.97	81.97	16.02.2012	40608	57888
17.02.2011	81.34	81.34	17.02.2012	40608	57888
18.02.2011	80.82	80.82	18.02.2012	40608	57888
19.02.2011	74.45	74.45	19.02.2012	40608	57888
20.02.2011	68.96	68.96	20.02.2012	40608	57888
21.02.2011	78.82	78.82	21.02.2012	40608	57888
22.02.2011	82.08	82.08	22.02.2012	40608	57888
23.02.2011	81.60	81.60	23.02.2012	40608	57888
24.02.2011	81.22	81.22	24.02.2012	40608	57888
25.02.2011	84.14	98.66	25.02.2012	40608	57888
26.02.2011	82.94	97.25	26.02.2012	40608	57888
27.02.2011	78.38	78.38	27.02.2012	40608	57888
28.02.2011	92.09	107.97	28.02.2012	40608	57888
01.03.2011	82.36	82.36	29.02.2012	40608	57888
02.03.2011	82.79	97.07	01.03.2012	44064	57888
03.03.2011	83.10	97.43	02.03.2012	44064	57888
04.03.2011	81.73	81.73	03.03.2012	44064	57888
05.03.2011	79.68	79.68	04.03.2012	44064	57888
06.03.2011	67.55	67.55	05.03.2012	44064	57888
07.03.2011	94.21	110.46	06.03.2012	44064	57888
08.03.2011	81.16	81.16	07.03.2012	44064	57888
09.03.2011	81.22	81.22	08.03.2012	44064	57888
10.03.2011	82.68	82.68	09.03.2012	44064	57888
11.03.2011	81.20	81.20	10.03.2012	44064	57888
12.03.2011	79.10	79.10	11.03.2012	44064	57888
13.03.2011	68.88	68.88	12.03.2012	44064	57888
14.03.2011	82.58	82.58	13.03.2012	44064	57888
15.03.2011	84.46	99.02	14.03.2012	44064	57888
16.03.2011	84.92	99.57	15.03.2012	44064	57888
17.03.2011	80.10	80.10	16.03.2012	44064	57888
18.03.2011	79.87	79.87	17.03.2012	44064	57888
19.03.2011	70.13	70.13	18.03.2012	44064	57888
20.03.2011	61.73	51.08	19.03.2012	44064	57888
21.03.2011	90.02	105.55	20.03.2012	44064	57888
22.03.2011	86.10	100.95	21.03.2012	44064	266976
23.03.2011	84.31	98.86	22.03.2012	44064	266976
24.03.2011	84.68	99.29	23.03.2012	44064	266976
25.03.2011	84.50	99.08	24.03.2012	44064	266976
26.03.2011	66.52	66.52	25.03.2012	44064	266976
27.03.2011	57.90	47.91	26.03.2012	44064	266976
28.03.2011	82.13	82.13	27.03.2012	44064	266976
29.03.2011	83.39	97.77	28.03.2012	44064	266976

30.03.2011	86.59	101.53	29.03.2012	44064	266976
31.03.2011	83.84	98.31	30.03.2012	44064	266976
01.04.2011	79.56	79.56	31.03.2012	44064	266976
02.04.2011	64.99	64.99	01.04.2012	76896	266976
03.04.2011	60.77	50.29	02.04.2012	76896	266976
04.04.2011	84.55	99.14	03.04.2012	76896	266976
05.04.2011	82.96	97.27	04.04.2012	76896	266976
06.04.2011	78.71	78.71	05.04.2012	76896	266976
07.04.2011	72.88	72.88	06.04.2012	76896	266976
08.04.2011	68.41	68.41	07.04.2012	76896	559872
09.04.2011	61.32	50.74	08.04.2012	76896	559872
10.04.2011	50.75	41.99	09.04.2012	76896	559872
11.04.2011	72.70	72.70	10.04.2012	76896	559872
12.04.2011	81.10	81.10	11.04.2012	76896	559872
13.04.2011	78.07	78.07	12.04.2012	76896	559872
14.04.2011	82.31	82.31	13.04.2012	76896	559872
15.04.2011	77.21	77.21	14.04.2012	76896	559872
16.04.2011	64.39	64.39	15.04.2012	76896	559872
17.04.2011	54.92	45.45	16.04.2012	76896	559872
18.04.2011	78.74	78.74	17.04.2012	76896	559872
19.04.2011	81.07	81.07	18.04.2012	76896	559872
20.04.2011	78.25	78.25	19.04.2012	76896	559872
21.04.2011	72.94	72.94	20.04.2012	76896	559872
22.04.2011	65.28	65.28	21.04.2012	76896	559872
23.04.2011	62.08	51.37	22.04.2012	76896	559872
24.04.2011	43.21	35.76	23.04.2012	76896	559872
25.04.2011	42.25	34.96	24.04.2012	76896	559872
26.04.2011	76.10	76.10	25.04.2012	76896	559872
27.04.2011	74.64	74.64	26.04.2012	76896	559872
28.04.2011	79.51	79.51	27.04.2012	76896	559872
29.04.2011	72.92	72.92	28.04.2012	76896	559872
30.04.2011	52.46	43.41	29.04.2012	76896	559872
01.05.2011	39.88	33.00	30.04.2012	76896	559872
02.05.2011	77.65	77.65	01.05.2012	266976	559872
03.05.2011	79.10	79.10	02.05.2012	266976	559872
04.05.2011	82.24	82.24	03.05.2012	266976	559872
05.05.2011	82.37	82.37	04.05.2012	266976	559872
06.05.2011	81.05	81.05	05.05.2012	266976	559872
07.05.2011	65.88	65.88	06.05.2012	266976	559872
08.05.2011	49.00	40.54	07.05.2012	266976	675648
09.05.2011	80.46	80.46	08.05.2012	266976	675648
10.05.2011	83.64	98.07	09.05.2012	266976	675648
11.05.2011	87.49	102.58	10.05.2012	266976	675648
12.05.2011	89.98	105.50	11.05.2012	266976	675648
13.05.2011	82.91	97.21	12.05.2012	266976	675648
14.05.2011	70.92	70.92	13.05.2012	266976	675648
15.05.2011	58.33	48.27	14.05.2012	266976	675648

16.05.2011	85.19	99.88	15.05.2012	266976	675648
17.05.2011	79.26	79.26	16.05.2012	266976	675648
18.05.2011	87.42	102.50	17.05.2012	266976	675648
19.05.2011	85.52	100.28	18.05.2012	266976	675648
20.05.2011	84.74	99.36	19.05.2012	266976	675648
21.05.2011	71.03	71.03	20.05.2012	266976	675648
22.05.2011	60.62	50.17	21.05.2012	266976	675648
23.05.2011	87.98	103.16	22.05.2012	266976	675648
24.05.2011	80.33	80.33	23.05.2012	266976	675648
25.05.2011	82.18	82.18	24.05.2012	266976	675648
26.05.2011	75.97	75.97	25.05.2012	266976	675648
27.05.2011	73.55	73.55	26.05.2012	266976	675648
28.05.2011	63.31	52.39	27.05.2012	266976	675648
29.05.2011	55.97	46.31	28.05.2012	266976	675648
30.05.2011	81.44	81.44	29.05.2012	266976	675648
31.05.2011	80.71	80.71	30.05.2012	266976	675648
01.06.2011	77.14	77.14	31.05.2012	266976	675648
02.06.2011	58.20	48.16	01.06.2012	559872	675648
03.06.2011	69.30	69.30	02.06.2012	559872	675648
04.06.2011	64.62	64.62	03.06.2012	559872	675648
05.06.2011	56.15	46.46	04.06.2012	559872	675648
06.06.2011	81.70	81.70	05.06.2012	559872	675648
07.06.2011	81.94	81.94	06.06.2012	559872	675648
08.06.2011	82.68	82.68	07.06.2012	559872	593568
09.06.2011	76.74	76.74	08.06.2012	559872	593568
10.06.2011	78.52	78.52	09.06.2012	559872	593568
11.06.2011	63.22	52.31	10.06.2012	559872	593568
12.06.2011	58.21	48.17	11.06.2012	559872	593568
13.06.2011	61.51	50.90	12.06.2012	559872	593568
14.06.2011	82.01	82.01	13.06.2012	559872	593568
15.06.2011	83.90	98.38	14.06.2012	559872	593568
16.06.2011	80.89	80.89	15.06.2012	559872	593568
17.06.2011	74.48	74.48	16.06.2012	559872	593568
18.06.2011	62.04	51.34	17.06.2012	559872	593568
19.06.2011	40.58	33.58	18.06.2012	559872	593568
20.06.2011	72.68	72.68	19.06.2012	559872	593568
21.06.2011	77.06	77.06	20.06.2012	559872	593568
22.06.2011	77.17	77.17	21.06.2012	559872	593568
23.06.2011	65.21	65.21	22.06.2012	559872	593568
24.06.2011	67.52	67.52	23.06.2012	559872	593568
25.06.2011	64.26	64.26	24.06.2012	559872	593568
26.06.2011	54.42	45.03	25.06.2012	559872	593568
27.06.2011	77.00	77.00	26.06.2012	559872	593568
28.06.2011	73.55	73.55	27.06.2012	559872	593568
29.06.2011	72.10	72.10	28.06.2012	559872	593568
30.06.2011	68.22	68.22	29.06.2012	559872	593568
01.07.2011	66.44	66.44	30.06.2012	559872	593568

02.07.2011	54.06	44.73	01.07.2012	675648	593568
03.07.2011	41.34	34.21	02.07.2012	675648	593568
04.07.2011	73.07	73.07	03.07.2012	675648	593568
05.07.2011	75.54	75.54	04.07.2012	675648	593568
06.07.2011	71.68	71.68	05.07.2012	675648	593568
07.07.2011	73.80	73.80	06.07.2012	675648	593568
08.07.2011	69.70	69.70	07.07.2012	675648	593568
09.07.2011	60.28	49.88	08.07.2012	675648	338638
10.07.2011	55.66	46.06	09.07.2012	675648	338638
11.07.2011	73.38	73.38	10.07.2012	675648	338638
12.07.2011	72.62	72.62	11.07.2012	675648	338638
13.07.2011	70.30	70.30	12.07.2012	675648	338638
14.07.2011	64.56	64.56	13.07.2012	675648	338638
15.07.2011	63.20	52.30	14.07.2012	675648	338638
16.07.2011	60.78	50.30	15.07.2012	675648	338638
17.07.2011	48.71	40.31	16.07.2012	675648	338638
18.07.2011	67.74	67.74	17.07.2012	675648	338638
19.07.2011	68.05	68.05	18.07.2012	675648	338638
20.07.2011	69.88	69.88	19.07.2012	675648	338638
21.07.2011	64.96	64.96	20.07.2012	675648	338638
22.07.2011	61.18	50.62	21.07.2012	675648	338638
23.07.2011	51.36	42.50	22.07.2012	675648	338638
24.07.2011	32.39	26.80	23.07.2012	675648	338638
25.07.2011	65.12	65.12	24.07.2012	675648	338638
26.07.2011	67.66	67.66	25.07.2012	675648	338638
27.07.2011	65.41	65.41	26.07.2012	675648	338638
28.07.2011	63.46	52.51	27.07.2012	675648	338638
29.07.2011	60.56	50.12	28.07.2012	675648	338638
30.07.2011	53.36	44.16	29.07.2012	675648	338638
31.07.2011	48.44	40.09	30.07.2012	675648	338638
01.08.2011	62.41	51.65	31.07.2012	675648	338638
02.08.2011	63.58	52.61	01.08.2012	593568	338638
03.08.2011	66.36	66.36	02.08.2012	593568	338638
04.08.2011	64.82	64.82	03.08.2012	593568	338638
05.08.2011	64.96	64.96	04.08.2012	593568	338638
06.08.2011	56.87	47.06	05.08.2012	593568	338638
07.08.2011	38.72	32.04	06.08.2012	593568	338638
08.08.2011	61.34	50.76	07.08.2012	593568	169344
09.08.2011	57.46	47.54	08.08.2012	593568	169344
10.08.2011	60.89	50.38	09.08.2012	593568	169344
11.08.2011	62.39	51.63	10.08.2012	593568	169344
12.08.2011	64.01	52.97	11.08.2012	593568	169344
13.08.2011	61.84	51.17	12.08.2012	593568	169344
14.08.2011	51.73	42.81	13.08.2012	593568	169344
15.08.2011	62.64	51.83	14.08.2012	593568	169344
16.08.2011	68.02	68.02	15.08.2012	593568	169344
17.08.2011	67.19	67.19	16.08.2012	593568	169344

18.08.2011	69.71	69.71	17.08.2012	593568	169344
19.08.2011	65.45	65.45	18.08.2012	593568	169344
20.08.2011	61.36	50.77	19.08.2012	593568	169344
21.08.2011	54.86	45.40	20.08.2012	593568	169344
22.08.2011	81.95	81.95	21.08.2012	593568	169344
23.08.2011	75.54	75.54	22.08.2012	593568	169344
24.08.2011	80.99	80.99	23.08.2012	593568	169344
25.08.2011	84.86	99.50	24.08.2012	593568	169344
26.08.2011	77.28	77.28	25.08.2012	593568	169344
27.08.2011	61.82	51.16	26.08.2012	593568	169344
28.08.2011	50.69	41.94	27.08.2012	593568	169344
29.08.2011	67.80	67.80	28.08.2012	593568	169344
30.08.2011	75.37	75.37	29.08.2012	593568	169344
31.08.2011	79.42	79.42	30.08.2012	593568	169344
01.09.2011	77.95	77.95	31.08.2012	593568	169344
02.09.2011	77.17	77.17	01.09.2012	338638	169344
03.09.2011	67.22	67.22	02.09.2012	338638	169344
04.09.2011	56.56	46.80	03.09.2012	338638	169344
05.09.2011	76.75	76.75	04.09.2012	338638	169344
06.09.2011	64.27	64.27	05.09.2012	338638	169344
07.09.2011	64.30	64.30	06.09.2012	338638	169344
08.09.2011	69.42	69.42	07.09.2012	338638	88992
09.09.2011	78.61	78.61	08.09.2012	338638	88992
10.09.2011	66.46	66.46	09.09.2012	338638	88992
11.09.2011	55.22	45.70	10.09.2012	338638	88992
12.09.2011	67.27	67.27	11.09.2012	338638	88992
13.09.2011	70.25	70.25	12.09.2012	338638	88992
14.09.2011	68.16	68.16	13.09.2012	338638	88992
15.09.2011	75.24	75.24	14.09.2012	338638	88992
16.09.2011	78.23	78.23	15.09.2012	338638	88992
17.09.2011	65.33	65.33	16.09.2012	338638	88992
18.09.2011	62.22	51.49	17.09.2012	338638	88992
19.09.2011	86.03	100.87	18.09.2012	338638	88992
20.09.2011	80.17	80.17	19.09.2012	338638	88992
21.09.2011	76.70	76.70	20.09.2012	338638	88992
22.09.2011	76.34	76.34	21.09.2012	338638	88992
23.09.2011	75.00	75.00	22.09.2012	338638	88992
24.09.2011	62.65	51.84	23.09.2012	338638	88992
25.09.2011	53.88	44.59	24.09.2012	338638	88992
26.09.2011	75.62	75.62	25.09.2012	338638	88992
27.09.2011	77.70	77.70	26.09.2012	338638	88992
28.09.2011	78.05	78.05	27.09.2012	338638	88992
29.09.2011	79.75	79.75	28.09.2012	338638	88992
30.09.2011	75.11	75.11	29.09.2012	338638	88992
			30.09.2012	338638	88992
